# Supra-pectineal quadrilateral buttress plating versus infra-pectineal plating in the management of quadrilateral plate fractures: A randomized controlled trial

**DOI:** 10.1007/s00264-024-06344-9

**Published:** 2024-10-16

**Authors:** Islam Sayed Moussa, Amr Mohammed Nagy

**Affiliations:** Department of Orthopedics and Traumatology, Faculty of Medicine, Ain Shams University, 56 Ramses Street, Abbasia, Cairo, 11522 Egypt

**Keywords:** Anatomical supra-pectineal quadrilateral plate, Infra-pectineal plating, Matta scoring system, Modified merle d'aubigne & Postel scoring system, Modified Stoppa approach, Quadrilateral plate fractures

## Abstract

**Purpose:**

Management of quadrilateral plate fractures is technically demanding and requires specific fixation techniques. Infra-pectineal plating is the gold standard method of fixation. However, we recorded a high incidence of medial wall displacement and reoperations. Therefore, the aim of our study was to identify whether supra-pectineal quadrilateral buttress plating provides much more rigid fixation with a better functional and radiological outcome or not.

**Patients and Methods:**

The authors conducted this prospective, randomized control, single-blinded study at a level 1 single trauma centre. Between March 2022 and June 2023, 34 patients with quadrilateral plate fractures had anterior fixation, either via the anatomical QLP (17 cases) or infra-pectineal plating (17 cases) (Groups A and B, respectively). The radiological and clinical outcomes, as well as residual medial wall displacement, were the primary outcomes.

**Results:**

The mean follow-up was 14.47 months in group A and 15.24 months in group B. In group A, the mean operative time (*p* = 0.02) was shorter, and the mean blood loss (*p* < 0.001) was significantly lower. However last follow-up showed no statistically significant differences as regards residual medial wall displacement (*p* = 1.0), final radiological (*p* = 0.86), and clinical outcomes (*p* = 1.0).

**Conclusion:**

Authors concluded that the anatomical QLP made it easier to reduce and fix acetabular fractures with a displaced medial wall. This was done by using multidirectional screws in the posterior column through its infra-pectineal extension and a strong screw purchase aimed at the posterior column through its supra-pectineal part**.** The two groups were similar in terms of final radiological and clinical outcomes, as well as residual medial wall displacement rates. However, the QLP had less morbidity than the classic infra-pectineal plating (shorter operation time and less blood loss).

## Introduction

The management of acetabular fractures that involve the quadrilateral plate is technically challenging [1]; the fracture pattern and configuration are difficult to allow for anatomical reduction of the displaced fragments [2]. To minimize the complications associated with immobilization, rigid fixation is required for fractures involving the quadrilateral plate [3]. Failure to obtain anatomical reduction in such injuries has led to high rates of reoperations and the need for hip arthroplasty [3].

The emergence of new pelvic implants has allowed for more stable and rigid fixation of the quadrilateral plate, even in severe comminution in young adults or severe osteoporosis in the elderly [4]. Researchers described applying long buttress screws through a suprapectineal plate along the medial wall of the acetabulum [5]. However, there is still no specific consensus regarding the optimal choice of anatomical plates for quadrilateral surface (QLS) fixation. The ilio-inguinal approach has been the gold standard to manage anteromedial acetabular fractures. This involves using a classis supra-pectineal plate and either a separate infra-pectineal plate or long periarticular buttress screws [6] to fix the medial wall. Recently, new designs came up with pre-bent plates with infra-pectineal extensions to buttress the quadrilateral surface. Also, with the widespread use of the anterior intrapelvic approach, the options for anatomical reduction and medial wall support have increased. The researchers previously used only a few of these implants with midterm and long-term outcomes [7].

The emergence of the modified stoppa approach allowed for wider exposure of the anteromedial acetabulum, also with the advantage of having less morbidity and not needing the additional middle window of the ilio-inguinal approach. Guerado et al. [8] recommend using the modified stoppa approach for anterior acetabular fractures that involve the quadrilateral plate. The infra-pectineal plating applied through the anterior intra-pelvic approach was considered the gold standard for the management of such injuries [9]. However, recent studies have shown that anatomical supra-pectineal quadrilateral buttress plating (QLP) is favourable for these types of injuries. One example is the study by Ramesh Ksen et al. [10], which demonstrated that this type of intervention is both effective and safe.

**Fig. 1:**
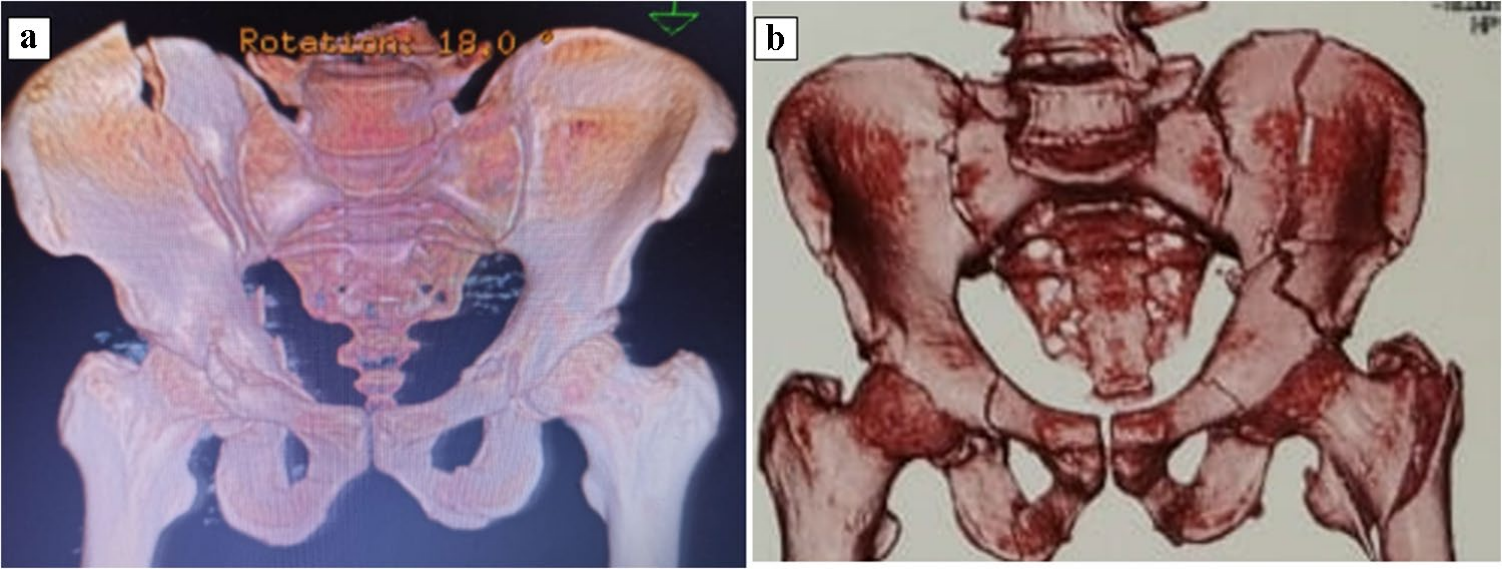
shows preoperative CT 3d that show: **a**-associated BC, and **b**- anterior column posterior hemi-transverse fracture acetabulum, respectively

The authors encountered an algorithm to determine whether the supra-pectineal QLP allows for a more rigid fixation with a better functional and radiological outcome than the classic infra-pectineal contoured plating for the fixation of quadrilateral plate fractures. We included only associated both column fractures with main displacement anterior and anterior column posterior-hemi transverse fractures (Fig. 1) and excluded fractures with associated pelvic ring injuries that require definitive fixation, internal organ injuries, and open fractures. We hypothesized that using the supra-pectineal QLP would provide much more rigid fixation and a strong buttress for medial wall migration, as well as a better functional and radiological outcome.

There is a lack of knowledge in the prospective assessment of functional and radiological outcomes and follow-up of postoperative complications of supra-pectineal quadrilateral buttress plating versus infra-pectineal plating in fixation of QLS, and to our knowledge, this will be the first study to include such measures of outcome together in a prospective randomized fashion. So, we conducted a prospective randomized controlled study (RCT) with the goal of reaching a satisfactory outcome and comparing supra-pectineal QLP versus infra-pectineal QLP for management of the anterior column with or without posterior hemi-transverse component and quadrilateral plate involvement.

## Patients and methods

After we obtained the Hospital Research/Ethics Committee approval (FWA 000017585 FMASU R66/2022), we conducted a RCT between March 2022 and June 2023 with clinical trial number NCT06440590. All patients signed a written informed consent. In order to enroll patients in the study, we conducted a thorough screening process that included a detailed clinical assessment of their medical history, a physical examination, and radiological investigations. This involved taking plain x-rays of the pelvis from different perspectives (AP, obturator, and iliac views) as well as conducting CT scans of the pelvis.

Two independent senior radiologists classified the injuries according to the Jude and Letournel classification. Our study included patients who met the selection criteria. 44 people between the ages of 16 and 60 were included in this study. They all had either associated bilateral column fractures (BC) or anterior column posterior-hemi transverse fractures of the acetabulum involving the quadrilateral plate. The study excluded patients with isolated posterior column or wall fractures; associated both column fractures that required posterior column fixation; open fractures; associated pelvic ring injuries that require intervention; patients less than 16 or older than 60 years’ old; and fractures exceeding two weeks.

**Fig. 2:**
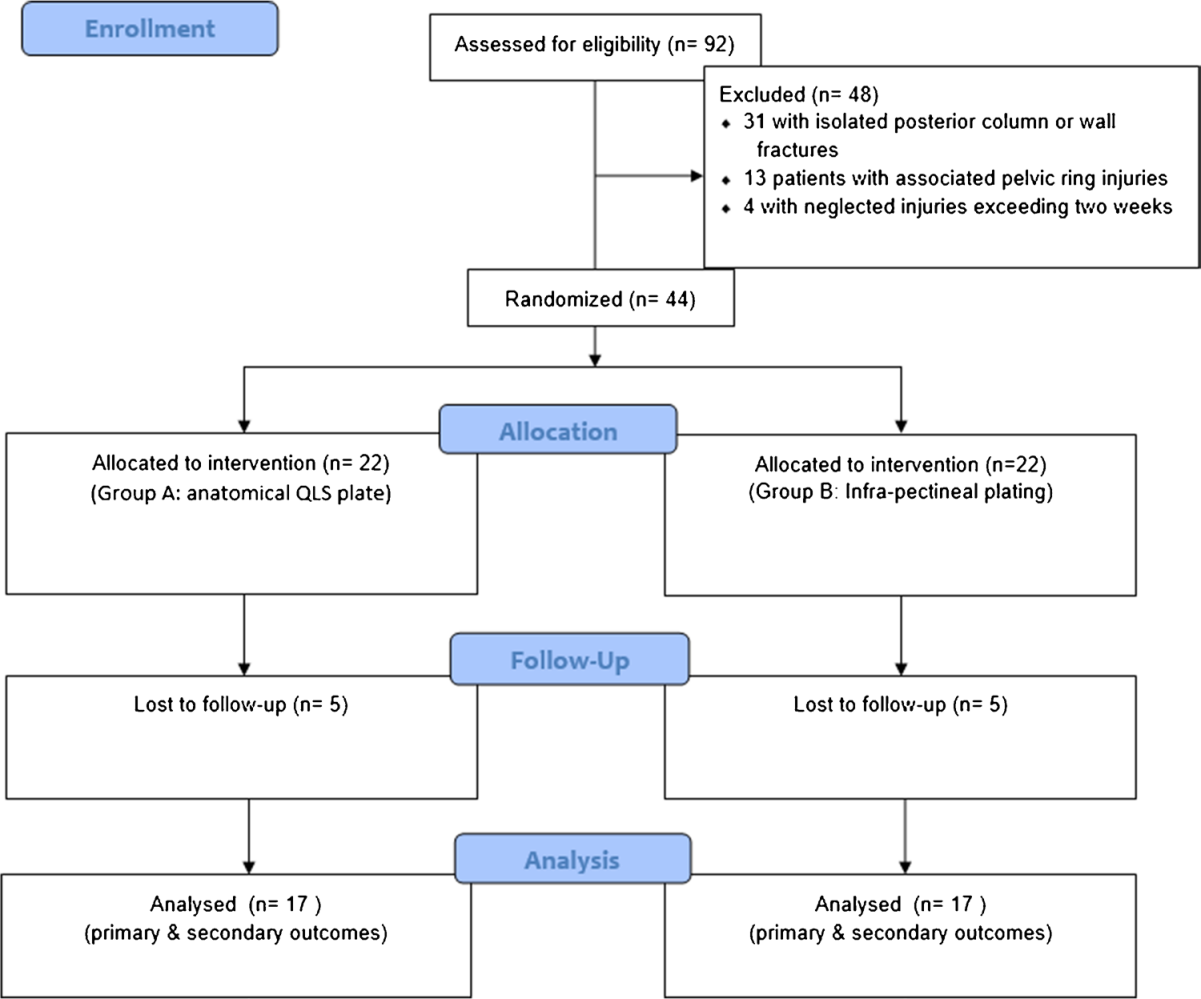
The study group's consort flow diagram

The authors created the randomization sequence using computerized numbers with a 1:1 allocation via random blocks two, four, six and eight. The irrelevant doctor assigned the sample numbers in an even distribution between the two groups; one group had a supra-pectineal QLP (group A), and the other had infra-pectineal plating (group B). Thirty-four patients (77%) in the analysis reached the final follow-up; we lost ten cases at follow-up (Fig. 2).

**Fig. 3:**
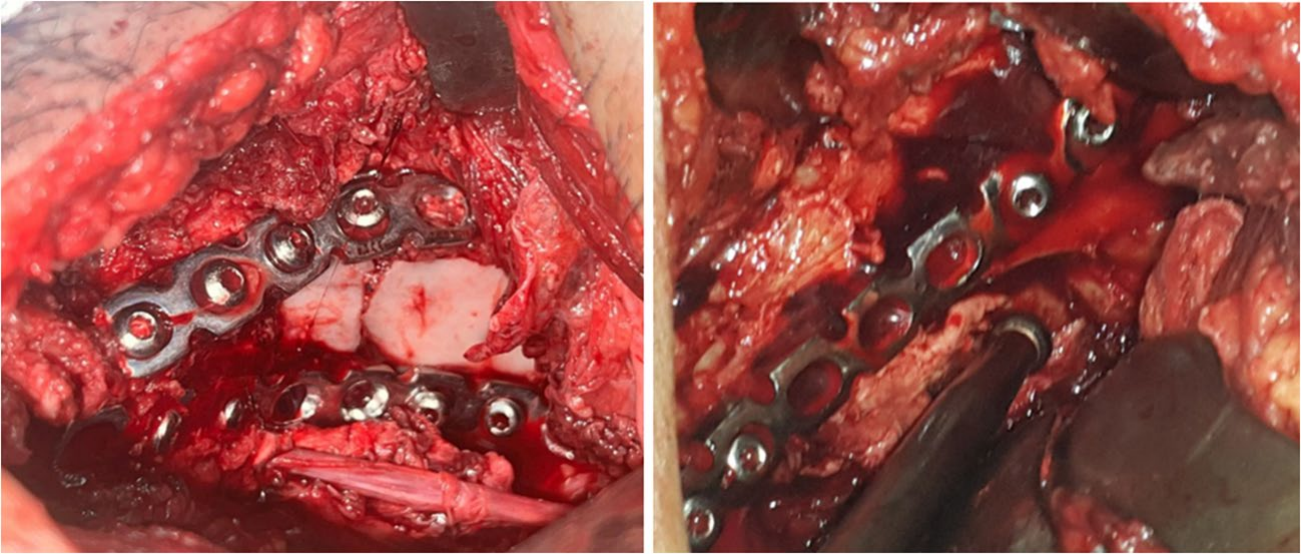
Intraoperative images for quadrilateral plate fracture reduction and fixation via supra- and infra-pectineal plates

The anterior intra pelvic approach, along with the lateral window of the ilioinguinal approach, were utilized in all cases. Following anatomical reduction, patients were categorized into two groups: group A comprised nine cases (53%) with a BC fracture and eight cases (47%) with an anterior column posterior hemi-transverse fracture, while group B had ten cases (59%) with a BC fracture and seven cases (41%) with an anterior column posterior hemi-transverse fracture. Group A received a standard 14-hole QLP, starting with an infra-acetabular screw and fitting the buttress part of the plate into the QLS, while group B received two plates, supra and infra-pectineal (Fig. 3), for the anterior column and the medial wall (Tables 1 and 2). After achieving anatomical reduction of the QLS and the central dislocation, BC fractures were checked intra-operatively for the accuracy of posterior column reduction in the iliac views intra-operative, and anterior to posterior screws were applied through the supra-pectineal plate. However, all cases that failed to achieve appropriate posterior column reduction and required separate sessions for posterior column fixation were excluded from the study.

**Table 1:** Comparison between two methods regarding demographic data of patients

		Supra-pectineal quadrilateral buttress plate (*N* = 17)				Infra-pectineal plate (*N* = 17)				t*	*P* value	
		Min	Max	Mean	SD	Min	Max	Mean	SD			
Age (years)		16.00	60.00	40.41	15.19	18.00	52.00	35.59	12.55	1.01	0.32	
		N		%		N		%		X^2**^	*P* value	
Sex	Male	7		41.2%		14		82.4%		6.10		0.01
	Female	10		58.8%		3		17.6%

**Table 2:** Comparison between two methods regarding pre-operative data:

		Supra-pectineal quadrilateral buttress plate (*N* = 17)		Infra-pectineal plate (*N* = 17)		X^2*^	*P* value
		N	%	N	%		
Side (according to posterior ring injury)	Right	8	47.1%	11	64.7%	1.07	0.30
	Left	9	52.9%	6	35.3%		
Classification of fractures	Associated both column fracture	9	52.9%	10	58.8%	0.12	0.73
	Anterior column posterior-hemi transverse	8	47.1%	7	41.2%		

*Chi square test

We encouraged both groups to follow the toe touch weight-bearing protocol [11] for six weeks, using axillary crutches to support 20% of their body weight for the injured limb. Every follow-up visit included x-rays of the pelvis, AP, obturator, and iliac views for all patients at 1.5 months, three months, six months, and one year after the surgery.

In week two, hip passive and active-assisted range of motion (ROM) were started, followed by an unassisted weight-bearing protocol with abductor muscles and quadriceps muscle strengthening in week six. Patients regained complete weight-bearing and full ROM, and they were able to return to work by the end of the third month. After six months and one year, the patient underwent a full radiological and clinical assessment and was able to resume their pre-injury mobility and athletic activities. [11]

Our RCT's research question was whether anatomical quadrilateral buttress plates improved clinical and radiological outcomes or not. The radiological and clinical outcomes, as well as residual displacement, were the primary outcomes. The secondary outcomes included other intra- and postoperative complications, mean blood loss, mean operative time, postoperative reduction assessment, amount of displacement, and the need for another operation.

We analyzed the post-operative reduction using Matta's description [11, 12], measuring the greatest displacement in the major fracture line in any of the three views, and assessing the residual displacement in the medial joint space in accordance with his description. The investigators categorized the patients into anatomic (0–1 mm), congruent (2–3 mm), and incongruent (greater than 3 mm) depending on the measured displacement on these radiographs.

All patients underwent radiological assessment using the Matta scoring system [11, 12], which included a plain x-ray pelvis showing both hips: anteroposterior (AP), obturator, and iliac views, as well as a CT pelvis if available. The investigators categorized the results into four categories: excellent (a hip joint that appears normal), good (minimal sclerosis and joint space narrowing less than 1 mm), fair (moderate sclerosis and joint space narrowing less than 50%), and poor (advanced arthritic changes). The final follow-up visit provided the best judgment and analysis of the interpretation from the anteroposterior view.

At the final follow-up, the investigators conducted a clinical assessment using the d'Aubigne & Postel score [13], analyzing it out of 18 and presenting the mean value. The score evaluates three factors: pain, mobility, and the ability to walk. The investigators categorized the patients into excellent (18), good (15–17), fair (12–14), and poor (3–11). The investigators followed the patients for a minimum of one year.

## Statistical analysis

We analyzed the data using the Statistical Program for Social Science (SPSS) version 20.0. The authors presented the quantitative data as the mean ± standard deviation (SD) and the qualitative data as frequency and percentage. We used the Epi Info 7 program to calculate the sample size, setting alpha error at 5% and power at 80%. Using the 11 program for sample size calculation, we determined that a sample size of at least 30 patients with quadrilateral plate fractures (15 patients undergoing supra-pectineal QLP and 15 patients undergoing infra-pectineal plating) will achieve a power of > 99% and can detect a statistically significant difference between the two groups in terms of the operation time in minutes: (109.9 + -33.6) and (236.1 + -97.9), respectively, according to Kwak et al. [14], 2022. This includes a 10% dropout rate and a 5% alpha error. Based on this, the adequate sample size was 30 total (15 cases per group).

This study was parametric, and we assumed normality of measured quantitative data because the sample size was greater than 30 and the standard deviation was small compared to the mean. The researchers used the student t-test to analyze the mean differences between the two groups and the Chi-square test to compare categorical variables. They set a 95% confidence interval with a 5% margin of error. Consequently, any *p*-value ≤ 0.05 was deemed statistically significant, with a *p*-value ≤ 0.001 (highly significant) and a *p*-value > 0.05 (insignificant).

## Results

Our study included 34 patients; the mean age was 40.4 years in group A and 35.6 years in group B (Table 1). In group A, we had nine patients with associated BC fracture (53%), eight patients with anterior column posterior hemi transverse fracture (47%), ten cases with associated BC fracture in group B (59%), and seven cases with anterior column posterior hemi transverse fracture (41%) (Table 2). In all cases, we used the modified Stoppa approach and the lateral window of the ilioinguinal approach to perform anatomical reduction and fixation according to the allocated group for each patient. The investigators observed no significant difference in baseline demographic data between the two groups (Tables 1 and 2).

The authors conducted an analysis of the primary outcomes and found no significant differences radiological assessment via the Matta scoring system [11, 12] between the two groups (Table 3); in group A, excellent in 76.5% of cases, good in 6% of cases, fair in 12% of cases, and poor in 6% of cases, while in group B, excellent in 70.6% of cases, good in 11.8% of cases, fair in 6% of cases, and poor in 11.8% of cases.

**Table 3:** Comparison between two methods regarding post-operative and follow up data

		Supra-pectineal quadrilateral buttress plate (*N* = 17)				Infra-pectineal plate (*N* = 17)				t*	*P* value
		Min	Max	Mean	SD	Min	Max	Mean	SD		
Amount of displacement (mm)		2.00	5.00	2.75	1.50	2.00	8.00	4.25	2.87	0.93	0.39
Final clinical assessment (modified merle d'aubigne & Postel scoring system)		10.00	18.00	15.71	2.31	10.00	18.00	15.82	2.56	0.14	0.89
follow up (months)		12.00	19.00	14.47	2.50	12.00	22.00	15.24	3.15	0.78	0.44
		N		%		N		%		X^2**^	*P* value
Postoperative assessment of reduction	Anatomical	13		76.5%		12		70.6%		0.54 FE	1.00
	Congruent	3		17.6%		3		17.6%			
	Incongruent	1		5.9%		2		11.8%			
Residual medial wall displacement	No	13		76.5%		13		76.5%		0.00 FE	1.00
	Yes	4		23.5%		4		23.5%			
Final radiological assessment (Matta scoring system)	Excellent	13		76.5%		12		70.6%		1.33 FE	0.86
	Good	1		5.9%		2		11.8%			
	Fair	2		11.8%		1		5.9%			
	Poor	1		5.9%		2		11.8%			
Need for another operation	No	16		94.1%		16		94.1%		0.00 FE	1.00
	Debridement once	1		5.9%		1		5.9%			
Final clinical assessment (modified merle d'aubigne & Postel scoring system)	Excellent	4		23.5%		5		29.4%		1.52 FE	1.00
	Good	9		52.9%		9		52.9%			
	Fair	3		17.6%		1		5.9%			
	Poor	1		5.9%		2		11.8%			

*Student t test **Chi square test (FE: Fisher Exact)

At the final follow-up, we conducted a clinical assessment using the modified Merle d'aubigne and Postel scoring systems [13] to compare the results between the two groups after a year. Group A's mean score was 15.71, while Group B's was 15.82. There were no statistically significant differences between the two groups (Table 3).

Regarding residual medial wall displacement, the results showed no significant differences between the two groups (Table 3), with each group having four cases, or 23.5% of the total. Also, Matta's [11, 12] postoperative assessment of reduction showed no statistically significant differences between the two groups. Group A rated it as anatomical 76.5% of the time, congruent 17.6% of the time, and incongruent 5.9% of the time. Group B graded it as anatomical in 70.6% of cases, congruent in 17.6% of cases, and incongruent in 11.8% of cases (Table 3).

**Fig. 4:**
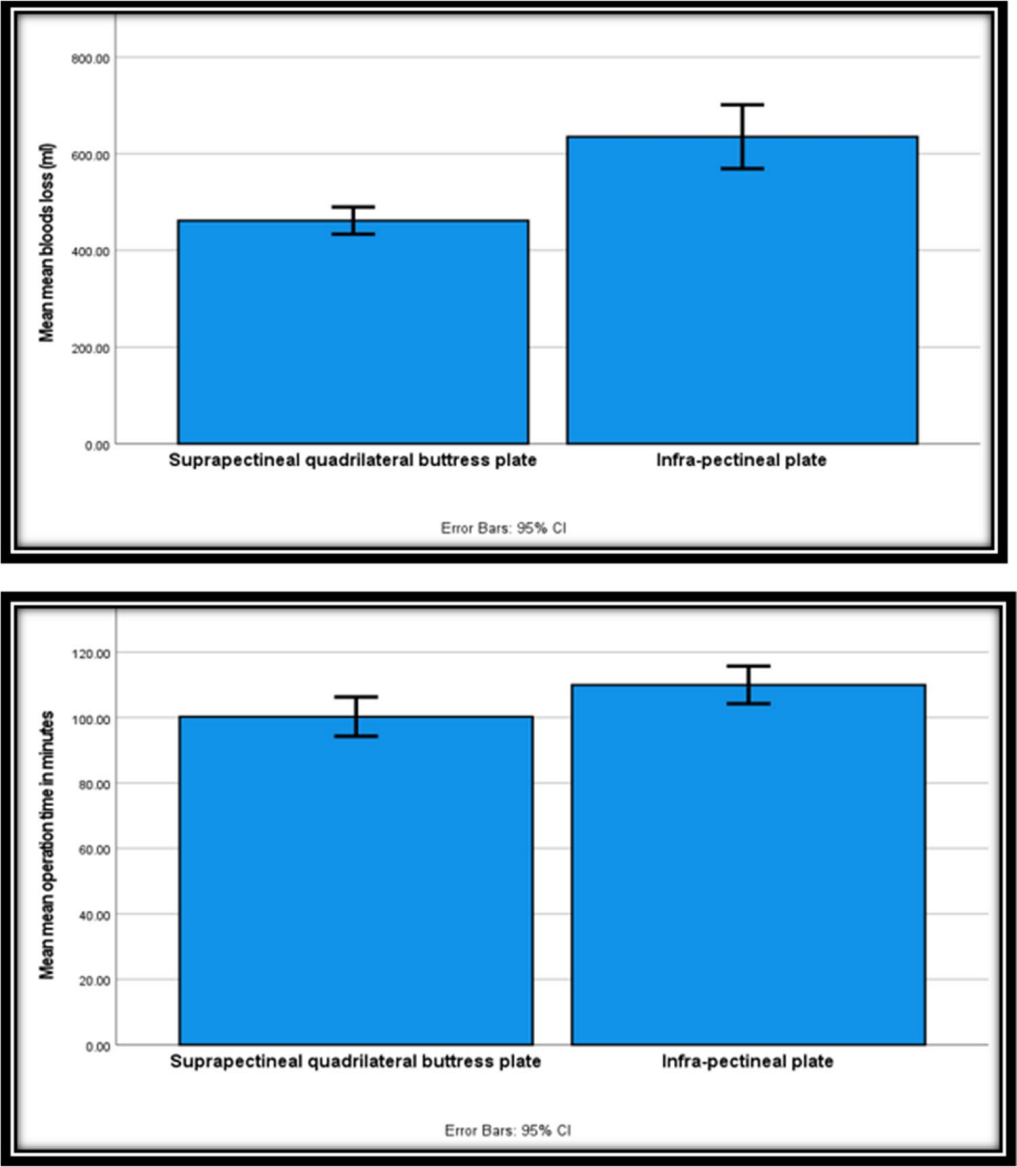
Error bar relation between mean operation time in hours and fixation principles (S) and between mean blood loss in mL and fixation principles (HS)

While the secondary outcomes analysis revealed that the mean operation time (Fig. 4) was significantly lower in group A (100.3 min) than in group B (110 min) (Table 4), the mean blood loss (Fig. 4) was significantly higher in group B (635.3 ml) compared to group A (461.7 ml), with statistically significant differences between the two groups (Table 4). Intra- and post-operative complications showed no statistically significant differences between the two groups, being slightly higher in group B (6 cases, 35.3%) than in group A (2 cases, 11.8%). Group A experienced wound infection in 5.9% of cases and limping with advanced arthritis in 5.9% of cases, whereas group B experienced obturator vein injury in 11.8% of cases, limping with advanced arthritis in 11.8% of cases, corona mortis injury in 5.9% of cases, and wound infection in 5.9% of cases (Table 4).

**Table 4:** Comparison between two methods regarding operative data:

		Supra-pectineal quadrilateral buttress plate (*N* = 17)				Infra-pectineal plate (*N* = 17)				t*	*P* value
		Min	Max	Mean	SD	Min	Max	Mean	SD		
Mean operation time in minutes		85.00	125.00	100.29	11.66	95.00	140.00	110.00	11.18	2.48	0.02
Mean bloods loss (ml)		400.00	600.00	461.76	54.57	400.00	950.00	635.29	128.41	5.13	< 0.001
		N		%		N		%		X^2**^	*P* value
Intra and postoperative complications	No	15		88.2%		11		64.7%		3.82 FE	0.49
	Obturator vein injury	0		0.0%		2		11.8%			
	Wound infection	1		5.9%		1		5.9%			
	Limping/advanced arthritis of hip joint	1		5.9%		2		11.8%			
	Corona mortis injury	0		0.0%		1		5.9%			
Intra and postoperative complications	No	15		88.2%		11		64.7%		2.62 FE	0.23
	Yes	2		11.8%		6		35.3%			

As regards the need for another operation, the investigators found no statistically significant differences between the two groups (Table 4); being equal in the two groups (Table 3), one case required debridement for wound infection once in each group (5.9%). Also, the mean amount of displacement showed no statistically significant differences between the two groups, with a mean value of 2.75 mm in group A and 4.25 mm in group B.

To summarize the results, the authors observed a highly statistically significant relationship between mean operative blood loss and fixation methods (Fig. 4), a statistically significant relationship between mean operation time in minutes and fixation methods (Fig. 4), and the infra-pectineal plating group showed longer operation time and greater intraoperative blood loss than the anatomical quadrilateral buttress plating group. However, we found no statistically significant correlation between the radiological outcome, the final clinical outcome, the mean clinical score, the radiological outcomes, the mean amount of displacement, the intra- and postoperative assessment of reduction, the residual medial wall displacement, the need for another operation, the intra- and postoperative complications, and the quadrilateral plate fixation method.

## Discussion

**Fig. 5:**
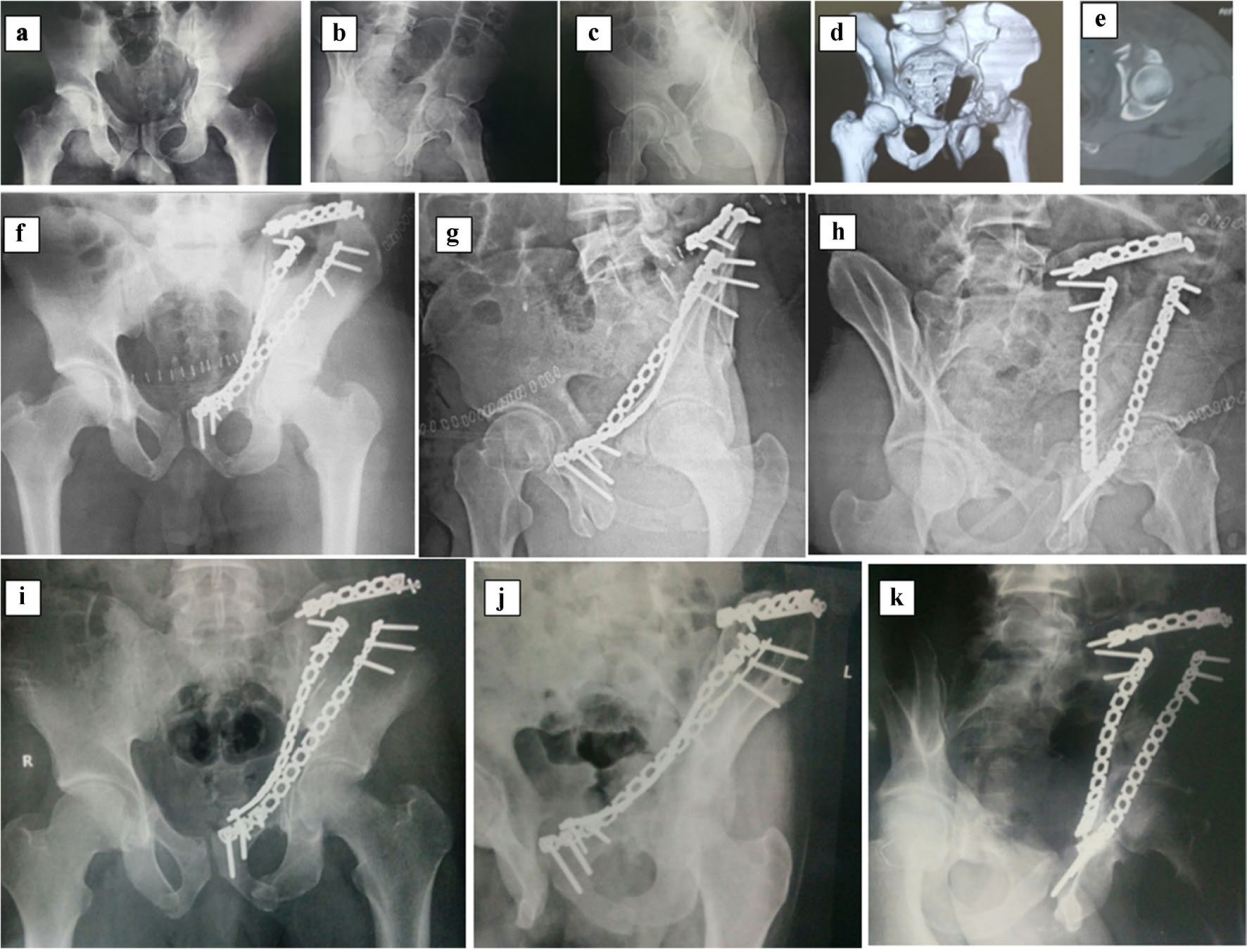
A 36-year-old RTA. **a**-**e** preoperative radiographs and computed tomography (CT) scan showing Left Associated Both Column fracture acetabulum. The **f**–**h** postoperative radiographs demonstrate the fixation via supra- and infra-pectineal plating and Iliac wing plate, while the **i**-**k** radiographs, taken 14 months postoperatively

**Fig. 6:**
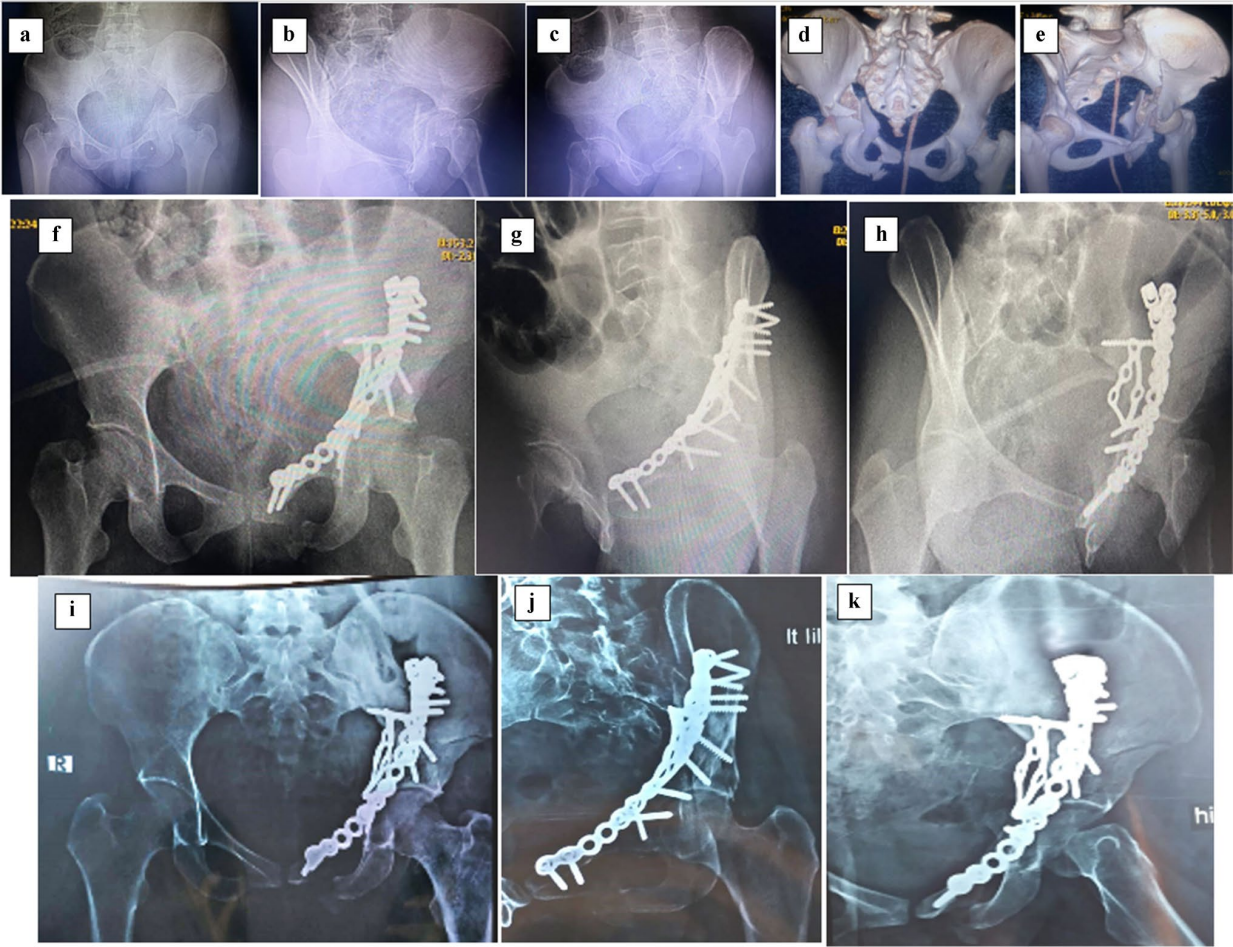
A 52-year-old female RTA. **a**-**e** preoperative radiographs and computed tomography (CT) scan cut showing Left Associated Both Column fracture acetabulum. **F**–**h** postoperative radiographs show fixation via anatomical QLP and iliac wing plating. **I**-**l** Radiographs taken 13 months postoperatively

The aim of our study was to compare supra-pectineal QLP versus infra-pectineal plating in the management of quadrilateral plate fractures. The outcomes for both groups were similar in terms of the radiological outcome (Figs. 5 and 6), the final clinical outcome, the mean clinical score, the radiological outcomes, the mean amount of displacement, the assessment of reduction during and after surgery, the need for another surgery, and complications during and after surgery. Group A showed a shorter operation time and less blood loss than Group B (Fig. 4).

Reduction and fixation of anteromedial displaced acetabular fractures remain challenging even for the most experienced surgeons. The goal of surgical treatment for quadrilateral plate injuries is to achieve anatomical reduction and stable fixation. [15] The gold standard method of fixation was infrapectineal buttress plating applied through the classic ilioinguinal approach; however, many surgeons now prefer the modified Stoppa approach that provides direct access medially to the quadrilateral plate, with the advantages of having less morbidity than the classic ilioinguinal approach, less blood loss, and no need for mobilization of the neurovascular bundle while gaining access through different windows. [16]

The emergence of anatomical pre-bent supra-pectineal QLP with infra-pectineal extension to buttress the medial wall helped the reduction and fixation of such fractures, especially in osteoporotic bones. The plate's inferior extension supports the anterior and posterior parts of the quadrilateral plate. [17] Additionally, applying infrapectineal plating with vigorous retraction of the urinary bladder and the obturator neurovascular bundle presents a technical challenge. Therefore, in our study, we utilized the modified Stoppa approach, which allows for wide exposure and direct visualization of the quadrilateral surface. We then began fixing the quadrilateral plate using supra-pectineal buttress plating and compared the results with those of classic infrapectineal plating. [17]

Our RCT consisted of 34 patients: (*n* = 17) formed the anatomical QLP group A, and (*n* = 17) formed the infra-pectineal plating group B. We found no significant differences in the demographic data between the two groups; additionally, we observed similar fracture patterns (*p* = 0.51), final clinical outcomes (*p* = 0.9), radiological outcomes (*p* = 0.8), and postoperative complications (*p* = 0.5). However, QLP group A showed significantly less blood loss (*p* < 0.001) and shorter operation time (*p* = 0.02), attributed to the shorter time needed to contour the infra-pectineal plate in group B and the lower risk of injury to the obturator vein.

After reviewing our study, we compared the reduction quality to the outcomes. According to Matta's description [11, 12], we found no significant differences between the two groups in the postoperative assessment of reduction (*p* = 1.0). Group A graded the reduction as anatomical in 76.5% of cases, congruent in 17.6% of cases, and incongruent in 5.9% of cases. In group B, the classification was anatomical in 70.6% of cases, congruent in 17.6% of cases, and incongruent in 11.8% of cases (Table 4). Achieving an accurate reduction would also lead to desirable final clinical and radiological outcomes. Our study outperformed Nicol et al. [18] retrospective study on 60 patients, grading the results as anatomical in 13% of cases, congruent in 23%, and poor in 43%.

The authors utilized the modified Merle d'aubigne and Postel scoring system [13], the final clinical outcome was comparable between the two groups (*p* = 0.89); the mean score at one-year follow-up was 15.71 in group A and 15.82 in group B (out of 18) (Table 4). The researchers also found that using pre-contoured anatomical QLP as a reduction tool makes it possible to directly support the medial wall. This lowers the risk of central dislocation and, as a result, ensures accurate reduction of the femoral head. Our study outperformed Meena et al. [11] and Karim et al. [5], both retrospective studies involving 90 and 40 patients, respectively. They compared the infra-pectineal plating via the ilioinguinal approach against the modified stoppa approach in quadrilateral plate fractures, achieving excellent or good grades in 68.13% and 88% of cases, respectively.

The radiological assessment via the Matta scoring system [11, 12] showed no significant differences (*p* = 0.86) between the two groups (Table 4); in group A, excellent in 76.5% of cases, good in 6% of cases, fair in 12% of cases, and poor in 6% of cases, while in group B, excellent in 70.6% of cases, good in 11.8% of cases, fair in 6% of cases, and poor in 11.8% of cases. Additionally, there were no significant differences in the residual medial wall displacement rates between the two groups (*p* = 1.0), with four cases in each group accounting for 23.5% of the total. Meena et al. [11] found similar results, grading 71.7% of cases as excellent or good and 28.3% as fair or poor.

The rates of intra- and post-operative complications were similar in the two groups (*p* = 0.23), slightly higher in group B (6 cases, 35.3%) than in group A (2 cases, 11.8%), especially obturator vein injury in two cases in group B. The need for another operation showed equal results (*p* = 1.0) between the two groups (Table 4); one case required debridement for wound infection once in each group (5.9%). Additionally, the mean amount of displacement showed no statistically significant differences (*p* = 0.4) between the two groups, with a mean value of 2.75 mm in group A and 4.25 mm in group B. The rates of intraoperative complications were comparable to the study by Meena et al. [11] (21%), and higher than that by Karim et al. [5] (17.5%); they mainly involved implant failure and obturator nerve palsy.

The anatomical QLP in group A showed a shorter mean operative time (100.3 min) than the infra-pectineal plating in group B (110 min), mainly due to the pre-contoured shape of the plate and the shorter time required for applying the anatomical plate. Also, the anatomical QLP in group A (461.7 ml) showed significantly less intraoperative blood loss (*p* < 0.001) compared to group B (635.3 ml), owing to the less need for vigorous medial retraction in group A and subsequently less risk of injury to the obturator bundle. (Fig. 3).

Our study had some limitations; the sample size was small, and the follow-up duration was short, attributed to the recent introduction of anatomical QLP at our centre. A performance bias may have been present because a single team performed all of the operations. Thus, later operations may have shown better results. In addition to incorporating two distinct types of acetabular fractures, we also excluded those that necessitated additional posterior column fixation. Despite all the limitations, this study was the first to compare such outcome measures together in a prospective random fashion between the two fixation groups to identify whether supra-pectineal quadrilateral buttress plating provides much more rigid fixation and a stronger buttress for medial wall migration, with subsequently better functional and radiological outcomes or not.

## Conclusion

The authors concluded that the anatomical QLP had a unique design that allowed easier reduction and fixation of acetabular fractures with a displaced medial wall, allowing for multidirectional screws in the posterior column through its infra-pectineal extension and also for strong screws purchase aiming the posterior column through its supra-pectineal part. The QLP also had less morbidity than the classic infra-pectineal plating (shorter operation time and less blood loss). The two groups were similar in terms of final radiological and clinical outcomes, as well as residual medial wall displacement rates. We plan to optimize the management of quadrilateral plate fractures in osteoporotic patients to improve radiological and clinical outcomes. Additionally, we aim to correlate the fixation method with the future need for arthroplasty conversion. To achieve this, we will conduct more future prospective studies with longer follow-up durations and a larger sample size.

## Data Availability

The authors confirm that the data supporting the findings of this study are available within the article [and/or] its supplementary materials.

## Abbreviations

QLP: Quadrilateral Buttress Plate
APC: Anteroposterior compression
BC: Both column
ROM: Range of motion
CI: Confidence interval
RCT: Randomized clinical trial
SD: Standard deviation

## Declarations

### Trial registration

Ain Shams University's ethical committee retrospectively registered and approved this trial. The guidelines of the International Council on Harmonization (ICH) in Anesthesiology and the Islamic Organization for Medical Sciences (IOMS) guided the organization and operation of the trial. The United States Office for Human Research Protections and the United States Code of Federal Regulations operate under Federal Wide Assurance No. 000017585 (retrospectively registered).
